# Extracellular vesicles in arbovirus infections: from basic biology to potential clinical applications

**DOI:** 10.3389/fcimb.2025.1558520

**Published:** 2025-04-28

**Authors:** Natalia Tiberti, Concetta Castilletti, Federico Giovanni Gobbi

**Affiliations:** ^1^ Department of Infectious, Tropical Diseases and Microbiology, IRCCS Sacro Cuore Don Calabria Hospital, Negrar di Valpolicella, Verona, Italy; ^2^ Department of Clinical and Experimental Sciences, University of Brescia, Brescia, Italy

**Keywords:** extracellular vesicles, arboviruses, biomarkers, pathogenesis, host-pathogen interaction

## Abstract

Arthropod-borne viruses, or arboviruses, are currently considered a global health threat responsible for potentially severe human diseases. The increased population density, changes in land use and climate change are some of the factors that are contributing to the spread of these infections over the last years. The pathogenesis of these diseases and the mechanisms of interaction with the host, especially those leading to the development of severe forms, are yet to be fully understood. In recent years extracellular vesicles (EVs) have emerged as important players in the inter-cellular and host-pathogen interaction arising a lot of interest also in the field of vector-borne viruses. In this context, EVs seem to play a dual role, by either promoting, thus facilitating, or preventing infection. Many studies are showing how viruses can hijack the vesiculation machinery to escape the host immune response and exploit EVs to sustain their replication and propagation, even though EVs shed by immune cells seem essential to promote antiviral responses. In this manuscript we reviewed the current knowledge regarding the association between EVs and vector-borne viruses, paying particular attention to their possible role in disease transmission and dissemination, as well as to their potential as novel tools for clinical applications, spanning from biomarkers of clinical utility to novel therapeutic options.

## 1 Introduction

Human infecting arboviruses represent a wide group of RNA viruses transmitted to the human host by blood-feeding arthropod vectors (i.e., mosquitos, ticks, sand flies) and responsible for significant human morbidity (Young, 2018; Goodman and Rasmussen, 2019). Arboviruses include viruses endemic in tropical and subtropical areas, i.e., Dengue virus (DENV), Zika virus (ZIKV), Chikungunya virus (CHIKV), yellow fever virus (YFV), Oropouche virus (OROV), Rift Valley fever virus (RVFV); as well as viruses also widespread in temperate regions, such as West Nile virus (WNV), Toscana virus (TOSV), Usutu virus (USUV), tick borne encephalitis virus (TBEV), Japanese encephalitis virus (JEV), Nairovirus (Crimean-Congo hemorrhagic fever (CCHFV)). Humans are incidental hosts for all of these viruses, except for DENV, for which they represent the primary host (Young, 2018).

Since the beginning of the 21^st^ century, we have been witnessing a significant spread of arboviral infections, associated with both the geographical expansion of the endemic areas and the increased number of local outbreaks in non-endemic regions (Gossner et al., 2018; Young, 2018; Franklinos et al., 2019; Huang et al., 2019; Young et al., 2021; Fortuna et al., 2024; Frasca et al., 2024). Factors contributing to these phenomena include increased population density, changes in land use and urbanization, climate change, global trade and travels (Caminade et al., 2019; Gibb et al., 2020; Chala and Hamde, 2021; Carlson et al., 2022; Thomson and Stanberry, 2022; Tan et al., 2024). All these elements impact both vector population dynamics and viral survival and behavior (Thomson and Stanberry, 2022).

Extracellular vesicles (EVs) are submicron membranous structures released by potentially all cell types and organisms, mainly under stress conditions or following cell activation. EVs represent a heterogeneous group of vesicular elements, which differ in both biogenesis and biophysical properties (van Niel et al., 2018). For long time they have been divided in two major groups: exosomes, i.e., smaller vesicles (30 – 150nm) originating within the multivesicular body (MVB) in the cell cytoplasm and released after fusion of the MVB with the cell membrane; and microvesicles (or microparticles), i.e., larger elements (100–1000 nm) released through a mechanism of budding of the plasma membrane (van Niel et al., 2018). However, due to the difficulties in distinguishing between the two populations and in specifically enriching only one or the other, it is now common practice to refer to these elements with the generic term EVs (Thery et al., 2018). In recent years, EVs have been arising a lot of interest for their unique properties as vehicles of biological information between cells or between organisms, being thus important players in the inter-cellular and cross-kingdom communication (Coakley et al., 2015). This attention has however been accompanied by the publication of a plethora of different methods for their enrichment, visualization and analysis, making in some instances data reproduction or study comparisons difficult. The approaches most commonly employed for EVs enrichment have been recently reviewed elsewhere (Clos-Sansalvador et al., 2022). As a consequence, the International Society for Extracellular Vesicles (ISEV) deemed necessary to harmonize the procedures and the criteria for investigating and reporting EVs studies, leading to the publication of the first guidelines in 2014, followed by updates in 2018 and 2023 (Lotvall et al., 2014; Thery et al., 2018; Welsh et al., 2024).

EVs are now well recognized players in many pathological processes, spanning from cancer, to neurodegenerative, cardiovascular and infectious diseases (Yates et al., 2022; Kumar et al., 2024). During pathological conditions, EVs are altered in size, number and content of biomolecules. All these properties make them a unique source of biomarkers as well as important tools to discover novel diagnostic or vaccine candidates. EVs can also functionally affect or alter recipient cells by delivering their cargo of biomolecules (particularly miRNA and proteins) or by triggering intracellular signaling cascades following interaction with cell surface receptors (van Niel et al., 2018).

**Figure 1:**
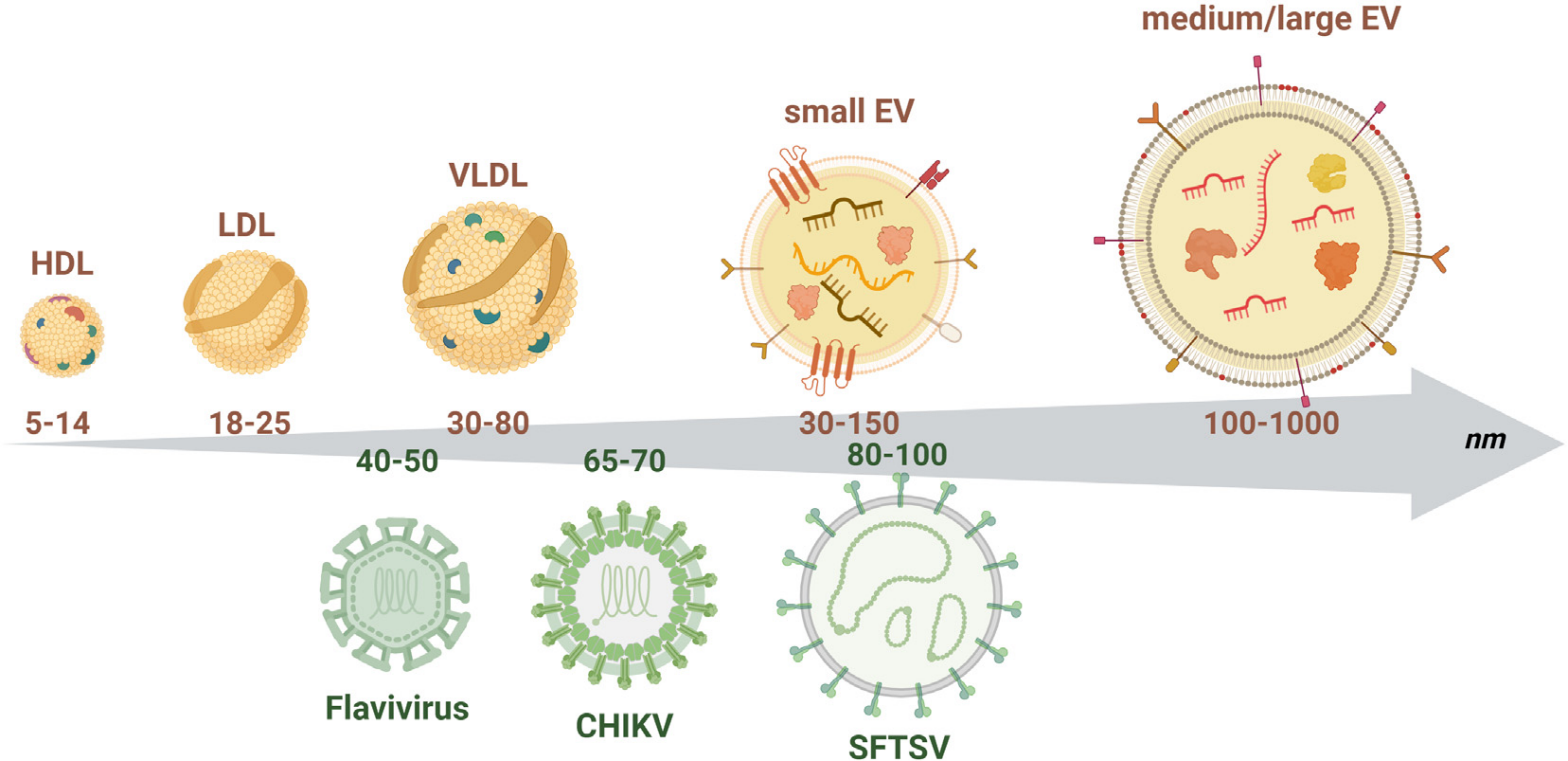
Schematic representation of the size distribution of extracellular vesicles and arboviruses. The upper part of the figure depicts small and medium/large EVs together with plasma lipoproteins (high, low and very low density lipoproteins - HDL, LDL, VLDL), which represent the main contaminants when enriching EVs from human plasma or serum. Small EVs are generally characterized by the presence of tetraspanins on their surface (i.e., CD63, CD9, CD81) while medium/large EVs by the exposure of phosphatidyl serine. Both types of vesicles enclose nucleic acids (small RNAs, mRNAs), proteins and lipids and carry on their surface different types of transmembrane proteins and receptors. The lower part of the chart represents the size of the different viral particles described in this review to highlight the important overlap in size with EVs. This overlap might represent an important issue when enriching EVs, since viral particles might be co-enriched. The numbers in the arrow represent the size range of the different particles in nm. Figure created in https://BioRender.com.

EVs and viruses share some important biological and physical properties – including size (**Figure 1**), which can make their investigation particularly challenging. The mechanisms of EVs biogenesis and of release of viral particles from infected cells present some common features. Indeed, viruses can be released from infected cells through a budding mechanism, similar to the one of release of microvesicles, or can form within the endosomal system involving the endosomal sorting complexes required for transport (ESCRT complex), as it occurs for exosomes (Nolte-’t-Hoen et al., 2016; Kutchy et al., 2020; Rey-Cadilhac et al., 2023). Moreover, the mechanisms of EVs uptake and viral entry also present similarities (Nolte-’t-Hoen et al., 2016).

Different viruses have been reported to hijack the EV biogenesis machinery to release virions or viral components (e.g., RNA, proteins), to support their propagation, to expand cellular tropism or to evade/modulate host immunity (Altan-Bonnet, 2016; Rey-Cadilhac et al., 2023). Viruses can in fact exploit the EV budding process i) to transfer viral material (in some cases the entire viral genome) (Nour and Modis, 2014; Altan-Bonnet et al., 2019); ii) as a mechanism of immune escaping, since viral RNA encapsulated within EVs is less immunogenic; iii) to reach immune privileged sites, as they can cross the blood-brain barrier (BBB) and the placental barrier (York et al., 2021). The first pieces of evidence indicating the presence of viral RNA within EVs released by infected cells have been collected in HIV, HTLV-1 and HCV (Chahar et al., 2015).

During viral infections, EVs can also be released by host immune cells, contributing to counteract the infection as they can trigger anti-viral responses and cytokine secretion (Martin et al., 2023), although such a mechanism could also be detrimental if it goes uncontrolled. The determinants regulating the ability of EVs to act as enhancers of the inflammatory process associated with the antiviral response or as mediators of immunosuppression are yet to be elucidated, even though timing seems to represent an important factor.

In light of their emerging role in viral infection and viral transmission, we believe that EVs might represent important factors to be taken into account not only to achieve a better understanding of the pathobiology of arboviral infections, but also to highlight novel alternative strategies to counteract the spread of these diseases. We thus deemed necessary to summarize the current state of the art regarding the association between EVs and vector-borne viruses. Particular attention will be paid to their possible role in disease transmission and dissemination, as well as to their potential as novel tools for clinical applications, spanning from biomarkers of clinical utility to novel therapeutic options.

## 2 Mosquito-borne viruses

### 2.1 Flaviviruses

#### 2.1.1 Dengue virus and dengue fever

Dengue fever is the most prevalent mosquito-borne viral infection worldwide (Hale, 2023). According to the WHO, half of the world’s population is currently considered at risk of infection (https://www.who.int/news-room/fact-sheets/detail/dengue-and-severe-dengue, last visited on December 4th 2024). As of December 2024, more than 7 million laboratory confirmed cases were reported by the WHO since January 2024, including more than 46000 severe cases and 9683 deaths (https://worldhealthorg.shinyapps.io/dengue_global/, visited on December 4th 2024), with the vast majority of the cases registered in the Americas.

Four different DENV serotypes (DENV1-4), sharing approximately 65% of their genome (Mishra et al., 2019), are responsible for the human disease. The main biological and clinical features of DENV infection are summarized in **Tables 1** and **2**. Secondary infection with a heterologous serotype is a well-established risk factor for the development of severe dengue (DHF) because of antibody dependent enhancement (ADE) (Mishra et al., 2019; Sawant et al., 2023); even though other patient- and virus-associated factors might contribute to disease evolution.

**Table 1:** Main biological features of the arthropod-borne viruses described in this manuscript.

Virus	Family	Genus	Vector	Permissive cells for *in vitro* growth	Genome and virion size
DENV	Flaviviridae	Flavivirus	*Aedes aegypti* *A. albopictus*	Monocytes, macrophages, DCs, platelets, lymphocytes, endothelial cells, epithelial cells, fibroblasts, neuronal cells	Single-strand, positive-sense RNA Virion: 40-50nm
ZIKV	Flaviviridae	Flavivirus	*Aedes aegypti* *A. albopictus* *Culex pipiens*	Immature neuronal progenitors, astrocytes, microglia, monocytes, macrophages, vascular endothelial cells	Single-strand, positive-sense RNA Virion: 50nm ca.
CHIKV	Togaviridae	Alphavirus	*Aedes aegypti* *A. albopictus*	Dermal fibroblasts, keratinocytes, melanocytes, endothelial cells, Langerhans cells, dendritic cells, monocytes, synovial macrophages	Single-strand, positive-sense RNA Virion: 65-70nm
WNV	Flaviviridae	Flavivirus	*Culex* spp. *Aedes koreicus*	Keratinocytes, neurons, glial cells, Langerhans cells, epithelial cells	Single-strand, positive-sense RNA Virion: 50nm ca.
JEV	Flaviviridae	Flavivirus	*Culex* spp.	Monocytes/macrophages, DCs, Microglia	Single-strand, positive-sense RNA Virion: 50nm ca.
Dabie bandavirus*	Phenuiviridae	Bandavirus	*Haemaphysalis longicornis*	HeLa, B cells, macrophages	Single-strand, negative-sense RNA Virion: 80-120nm

*Also known as Severe fever with thrombocytopenia syndrome (SFTS) virus. DC, dendritic cells.

**Table 2:** Main features of the clinical presentation of arboviral infections described in this manuscript.

Disease	Symptoms of mild infection	Symptoms of severe infection	Vaccine available	Laboratory diagnosis
Dengue fever	Fever, headache, retro-orbital pain, myalgia, arthralgia, rash	Dengue hemorrhagic fever/dengue shock syndrome: increased vascular permeability, hypovolemia, hypotension, shock, thrombocytopenia. Can affect the CNS causing neurological clinical manifestations	Yes	RT-PCR NS1 antigen Serology Neutralization assay
Zika infection	Maculopapular rash, with or without mild fever, arthralgia, fatigue, conjunctivitis/conjunctival hyperemia, myalgia, headache	Guillain-Barré syndrome, microcephaly and other CNS malformations (fetus)	No	RT-PCR Serology Neutralization assay
West Nile infection	Fever with headache, body aches, joint pain, rash	Meningitis, encephalitis and acute flaccid paralysis (WNND)	No	RT-PCR Serology Neutralization assay
Japanese encephalitis	Fever, diarrhea, headache, tiredness, nausea, vomiting	Reduced consciousness, seizures (particularly in children), parkinsonian syndrome, encephalitis. Features typical of meningitis may also be present.	Yes	RT-PCR Serology Neutralization assay
Tick-borne encephalitis	Fever, fatigue, myalgia, headache, nausea (during the first viremic phase)	Aseptic meningitis, meningo-encephalitis, myelitis, paralysis, radiculitis (second phase)	Yes	RT-PCR Serology Neutralization assay
Chikungunya infection	Fever, myalgia, headache, nausea, photophobia, joint pain, rash	Symmetric arthralgia, neurological, hemorrhagic and ocular manifestations, myocarditis, hepatitis, rare meningoencephalitis	Yes	RT-PCR Serology Neutralization assay
Dabie bandavirus* infection	Fever, gastrointestinal symptoms, lymphadenopathy	Hemorrhagic manifestations, encephalopathy, acute respiratory distress syndrome, multiple organ dysfunction	No	RT-PCR Serology Neutralization assay

*Also known as Severe fever with thrombocytopenia syndrome (SFTS) virus. DC, dendritic cells; CNS, central nervous system; RT-PCR, reverse transcription polymerase chain reaction; NS1, DENV non-structural protein 1; WNND, West Nile neuroinvasive disease.

From a pathophysiological perspective, DHF is associated with endothelial cell dysfunction and cytokine storm (i.e., uncontrolled production of pro-inflammatory cytokines), leading to increased vascular permeability/damage and plasma leakage (Malavige and Ogg, 2017). These severe manifestations are likely to be associated with the inflammatory response rather than with the virus itself, as they usually occur after viral clearance (Mishra et al., 2019).

##### 2.1.1.1 EVs and DENV pathogenesis

**Figure 2:**
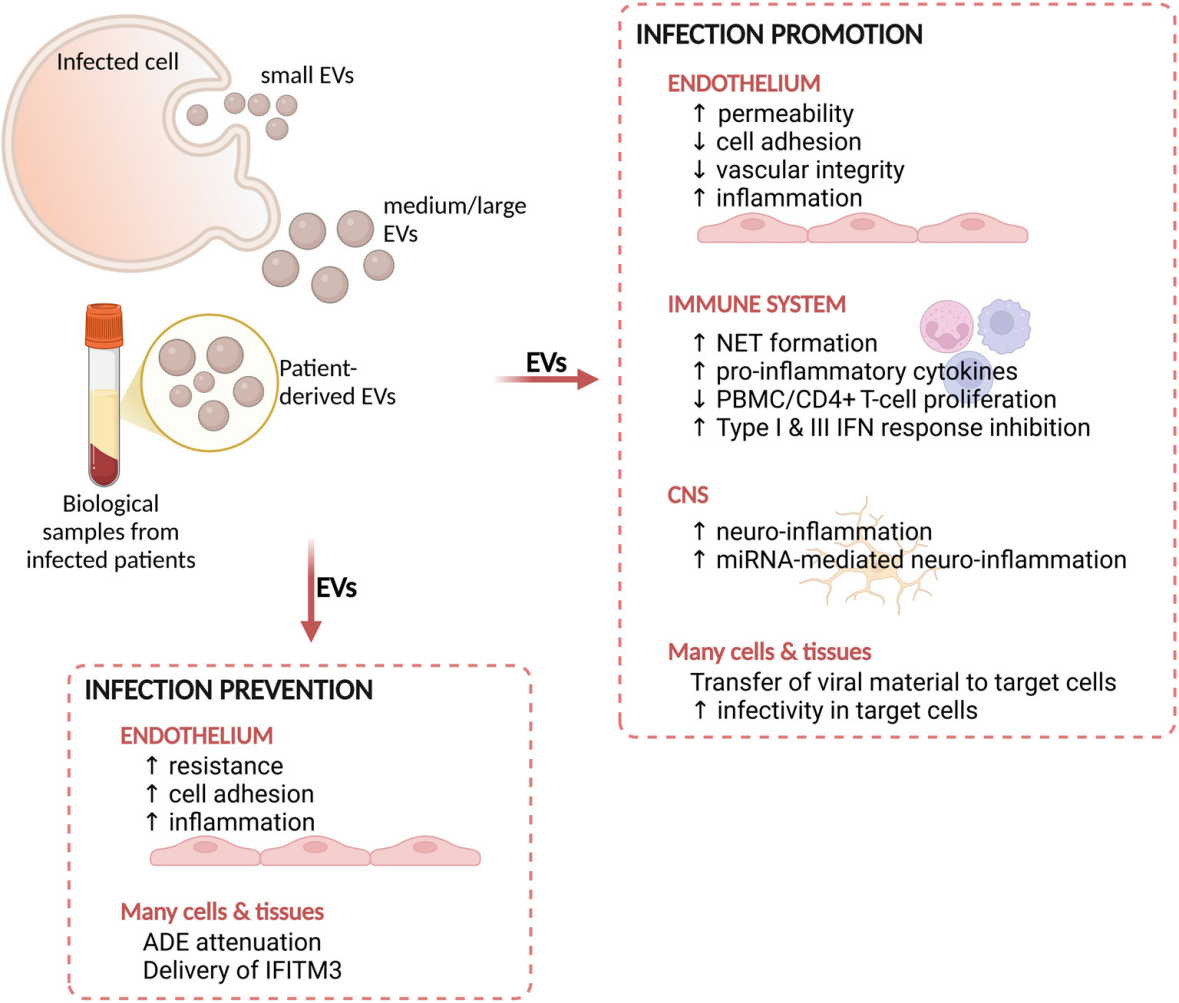
Summary of the main effects mediated by EVs during arboviral infections. EVs derived from cells infected *in vitro* or from biological samples from infected patients display functional effects on host cells or tissues. These effects can either promote the infectivity of target cells or prevent the infection. NET, neutrophil extracellular trap; PBMC, peripheral blood mononuclear cells; CNS, central nervous system; IFN, interferon; ADE, antibody dependent enhancement; IFITM3, interferon-inducible transmembrane protein 3. Figure created in https://BioRender.com.

Different cell types are activated during DENV infection, either as direct target of the virus, or as involved in the host response to the infection, representing potential sources of EVs (Khanam et al., 2022). Endothelial dysfunction and plasma leakage are key events leading to the development of severe dengue (Hottz et al., 2013b; Malavige and Ogg, 2017; Vedpathak et al., 2023). It is thus not surprising that, in the attempt to elucidate the functional role of EVs in DENV pathogenesis, many published studies focused on the interaction between EVs and endothelial or immune cells *in vitro* (**Figure 2**; **Supplementary Table S1**).

*In vitro*, platelets exposed to DENV2 released EVs containing IL-1β, a pro-inflammatory cytokine associated with severe dengue (Hottz et al., 2013a). These EVs increased human microvascular endothelial cell permeability, suggesting a potential functional role for IL-1β-rich EVs in the dysregulation of vascular permeability (Hottz et al., 2013a). The ability of platelet-derived EVs to alter endothelial integrity was also confirmed with platelets from DENV patients (Vedpathak et al., 2023), since EVs from severe dengue subjects, but not those from healthy controls, induced a significant loss in adhesion molecules and integrity of vascular endothelial cells. The same vesicles also increased the expression of vascular inflammation markers (namely CRP, SAA, sVCAM-1 and sICAM-1) in endothelial cells (Vedpathak et al., 2023). EVs from DENV-infected platelets released via C-type lectin receptor 2 (CLEC2) were also reported to affect innate immune cells. Indeed, they induced the activation of neutrophils and macrophages, leading to the formation of neutrophil extracellular trap (NET) and to the release of pro-inflammatory cytokines (Sung et al., 2019). Since uncontrolled inflammation and immune activation are phenomena associated with the development of severe dengue (Khanam et al., 2022), it could be hypothesized that platelet-derived EVs are important triggers of this inflammatory process.

Other cell types investigated for their ability to release EVs following DENV infection include hepatocellular carcinoma epithelial cells (HepG2) and umbilical vascular endothelial cells (HUVEC). Both cell lines were reported to release increased amount of Annexin V positive (AnV^+^)-EVs upon infection with DENV2 compared to mock-treated cells. Importantly, one third of these EVs harbored viral antigens (non-structural protein 1 (NS1) or envelope protein (E) or both) on their surface (Punyadee et al., 2015). It is however yet to be elucidated whether these antigens associated with EVs surface during EVs biogenesis or the positive signal was due to co-enrichment with EVs.

Functional studies were also performed ex vivo analyzing EVs circulating in patients’ blood (**Figure 2**). EVs from severe patients, carrying pro- and anti-inflammatory cytokines, mediated the suppression of cell proliferation in PBMCs and CD4^+^ T *in vitro* through PD-L1/PD-1 interaction, and induced changes in the frequencies of CD4^+^ T cell subsets towards a CXCR3^+^/CCR6^+^ phenotype (Kumari et al., 2023).

EVs might be involved in the neuro-inflammation associated with dengue (**Figure 2**), as already shown for other infectious conditions (Ruan et al., 2021; Wu et al., 2024). Mishra and colleagues explored this aspect by investigating the effects of EVs released by DENV2-infected monocytes on human microglia (Mishra et al., 2020). Infected THP1 and DENV-NS1-transfected HEK293T cells released EVs in the conditioned medium containing increased amount of miR-148a. When delivered to microglial cells, this miR-148a^+^-EVs induced the down-modulation of the ubiquitin-specific peptidase 33 (USP33)/ATF3 axis, a negative regulator of neuro-inflammation (Mishra et al., 2020).

As presented in the introduction, EVs can also participate to the antiviral defense against invading viruses (Martin et al., 2023) (**Figure 2**). Type I IFN represents an important mechanism of antiviral defense protecting cells from viral entrance (Mesev et al., 2019). Interestingly, it has been shown that EVs might contribute to the propagation of this IFN-mediated antiviral mechanism during HBV and HCV infections in different models (Li et al., 2013). EVs released from IFNα-treated PBMCs displayed antiviral effects on DENV3 infected cells, acting as a mechanism for immune cells to counteract viral replication and infection (Martins et al., 2018). Moreover, the delivery via EVs of interferon-inducible transmembrane protein 3 (IFITM3) - a potent inhibitor of viral entry in target cells - to different types of naïve cells prevented DENV infection (Zhu et al., 2015).

Experimental data are supporting the presence of viral proteins within EVs or associated with EV surface. *In vitro* experiments showed that DENV2 infected macrophages release EVs containing NS3 protein, which increased endothelial resistance, expression of adhesion molecules and the release of pro-inflammatory cytokines in endothelial cells (Velandia-Romero et al., 2020). Notably, NS3^+^-EVs were not infectious in epithelial cells (monkey kidney). These observations suggest that infected macrophages release EVs that activate the endothelium towards the establishment of an early protective pro-inflammatory response. More in depth investigations are however required to better clarify a number of aspects, including the timing of this immune-modulation and the effects on other cell types, including immune cells.

##### 2.1.1.2 EVs as DENV severity biomarkers

From a clinical perspective, the possibility of predicting the evolution towards severe dengue would represent a major breakthrough in patients’ management. EVs are thus being investigated as potential prognostic markers for severe dengue (DHF) (**Table 3**). EV-based liquid biopsies are currently regarded as promising tools for diagnostic or prognostic applications, even though both pre-analytical and analytical processes strongly need standardization before they can be translated into clinical practice (Irmer et al., 2023). Easily accessible body fluids like blood, urine or semen carry EVs, the quantitative alteration of which can be indicative of a pathological state. Different studies have attempted to quantify and compare circulating EVs during DENV infection. Patients with DHF were reported to harbor increased circulating CD41a^+^ platelet-derived EVs and increased pro- and anti-inflammatory cytokines in both blood and circulating EVs compared either to mild-dengue (DF), other febrile illnesses or healthy controls (Kumari et al., 2023), in agreement with previous reports (Hottz et al., 2013a). In contrast, the number of AnV^+^/CD41a^+^ EVs during the febrile illness was significantly lower in DHF patients compared to both DF and other febrile illnesses, likely because of thrombocytopenia (Punyadee et al., 2015), as well as in dengue patients (with or without warning signs) compared to healthy controls (Patil et al., 2018). These discrepancies might be associated with the type of EV population analyzed. Indeed, Punyadee and Patil investigated vesicles double positive for AnV (thus expressing phosphatidylserine on their surface) and CD41a in platelet poor plasma (Punyadee et al., 2015; Patil et al., 2018), while Kumari measured double positive CD63^+^/CD41a^+^ EVs pre-enriched by ultracentrifugation (Kumari et al., 2023). This highlights how sample preparation and pre-processing can affect the observations of experimental studies and supports the importance of a good-reporting practice in studies dealing with EVs, as also recommended by ISEV (Welsh et al., 2024).

**Table 3:** Summary of the most relevant findings regarding EVs isolated from human body fluids and their potential as biomarkers or treatment strategies for DENV or ZIKV.

EVs source	Type of donor	EV enrichment method	EV surface phenotype	EV cellular origin	Principal observations	Ref.
DENV						
Plasma	DENV patients	UC	CD63^+^/CD41a^+^	PLT	Increased in DHF pts vs. DF, vs. OFI or vs. controls Suppression of PBMCs and CD4^+^ T-cell proliferation Induction of CD4^+^ apoptosis Increased release of TNFα, IFNγ, IL-13 by CD4^+^ T-cell	(Kumari et al., 2023)
PPP	DENV patients	nd	AnV^+^/CD41a^+^	PLT	Decreased in DHF patients vs. DF or vs. OFI, during the acute phase	(Punyadee et al., 2015)
PPP	DENV patients	nd	AnV^+/^CD235a^+^	RBC	Increased in DHF vs. DF or vs. OFI during the acute phase	(Punyadee et al., 2015)
PPP	DENV patients	nd	AnV^+^/CD235^+^/NS1^+^	RBC	Increased in DHF vs. DF or vs. OFI during the acute phase	(Punyadee et al., 2015)
PPP	DENV patients	nd	AnV^+/^CD235a^+^	RBC	Increased in dengue patients with WS vs. without WS between day 4–7 from fever onset	(Patil et al., 2018)
Serum	DENV patients	ExoQuick	nd	na	Increased miR-96-5p and decrease miR-146-5p in severe dengue	(Pradhan et al., 2023)
ZIKV						
Semen	Healthy subjects	filtration-ultrafiltration	nd	na	EVs with size 50 - 400nm. Semen- EVs inhibit ZIKV infection in cells of the anogenital tract	(Muller et al., 2018)
Semen	Healthy subjects	Filtration + UC + DG	nd	na	Semen-EVs inhibit ZIKV infection in genital epithelial primary cells	(Wang et al., 2020)
Saliva, Urine, Vaginal lavage	Healthy subjects	Filtration-ultrafiltration + SEC	CD9, CD63, CD81, CD14, CD24, CD44, CD133, CD142, CD326	na	Inhibition of ZIKV infection in permissive cells Salivary EVs prevent viral attachment to human and simian target cells, thus inhibiting infection in a more potent fashion compared to EVs from other matrices	(Conzelmann et al., 2020)

UC, ultracentrifugation; PPP, platelet poor plasma; AnV, annexin V; DG, density gradient; SEC, size exclusion chromatography; PLT, platelets; RBC, red blood cells; PBMCs, peripheral blood mononuclear cells; nd, not done; na, not available.

Interestingly, RBC-derived EVs were suggested as potential predictive markers of severe dengue. AnV^+^/CD235^+^ RBC-EVs significantly raised in DHF patients as soon as 1 day before defervescence, suggesting that RBC-EVs could serve as early biomarkers for plasma leakage (Punyadee et al., 2015). DENV-patients with warning signs presented significantly higher RBC-EVs (AnV^+^/CD235a^+^), starting at day 4 of infection (Patil et al., 2018). As already shown *in vitro* (Punyadee et al., 2015), some of these EVs were also associated with viral antigens, namely E and NS1 proteins, with AnV^+^/CD235^+^/NS1^+^-EVs being significantly more abundant in severe patients (Punyadee et al., 2015). The association with RBC-derived EVs and DENV severity should now be investigated in a multi-center cohort of patients in order to verify their potential as severity markers and pave the way for clinical investigations.

DENV NS1 protein is detected in patients’ blood during acute infection and is currently used as a diagnostic tool (Frazer and Norton, 2024). Similarly to host TNFα and IL-1β, NS1 was reported to be involved in the vascular leakage (Hottz et al., 2014; Modhiran et al., 2015; Inyoo et al., 2017), even though its functional role has yet to be fully elucidated. The current knowledge indicates that NS1 can exert different biological functions, including i) promotion of the formation of vesicle pockets hosting the viral replication machinery; ii) interaction with the immune system and immune evasion; iii) mediation of alteration of endothelial integrity (Safadi et al., 2023). The association of NS1 molecules with EVs from DENV2 infected cells is particularly intriguing and supports a role for EVs as conveyors of this viral toxin to target cells contributing to dengue pathogenesis (Safadi et al., 2023), even though it cannot be excluded that biomolecules other than NS1 might be responsible for the functional effects mediated by EVs.

Dendritic cells (DCs) are another important cell type involved in the early phases of DENV infection and their dysfunction might be associated with disease severity (Schmid et al., 2014; Khanam et al., 2022). DCs act as antigen-presenting cells with phagocytic properties, representing a link between the innate and the adaptive immunity. They are known to secrete cytokines and EVs with immunomodulatory functions (Shenoda and Ajit, 2016). Human primary DCs were reported to display an altered EVs cargo of miRNA and mRNA upon infection with mild or severe DENV3 strains (Martins et al., 2018), with the latter also triggering a strong cytokine release (Silveira et al., 2011). EVs from infected cells carried strain-specific or DENV-specific miRNA (Martins et al., 2018), some of which (i.e., let-7e, miR-1261, miR-142, miR-371b, and miR-4327) had already been proposed as associated with DENV infections (Tambyah et al., 2016). Moreover, EVs from severe DENV3-DCs contained mRNA involved in DC immune-activation pathway, which was instead inhibited in EVs from mild-DENV DCs, supporting the hypothesis that the virus might exploit the vesiculation route to sustain viral propagation (Martins et al., 2018).

The detection of EVs cargo specific of different strains or disease states highlights the importance of EVs as sources of novel candidate prognostic markers. Alterations in the biomolecular content of circulating EVs in infected patients have also been shown as associated either with the disease state (i.e., compared to healthy controls) or with disease progression. EVs were in fact shown to carry miRNAs species, the abundance of which was significantly increased (miR-96-5p) or decreased (miR-146-5p) during disease progression, indicating their potential utility as early prognostic markers (Pradhan et al., 2023), although formal validation is yet to be performed.

##### 2.1.1.3 EVs during DENV transmission

EVs might also be involved in DENV transmission at the time of mosquito bite and feeding. Vector-derived EVs might thus be of particular interest to understand – and potentially alter – the mechanisms of infection transmission.

EVs released from DENV-infected C6/36 cells (*A. albopictus* cell line) have been reported to contain viral RNA and proteins. Importantly, these EVs were also reported to carry the full-length viral genome and to be able to infect mosquito and human (keratinocytes and endothelial cells) naïve cells *in vitro* (Vora et al., 2018). Similarly, small EVs CD9^+^ released by DENV2-infected C6/36 cells displayed larger size compared to EVs released by mock-treated cells, contained virus-like particles and were able to infect naïve-mosquito cells (Reyes-Ruiz et al., 2019). These observations agreed with previous data (Vora et al., 2018) but, through the detection of virus-like particles enclosed within EVs, suggest the vesicular pathway as an alternative route for viral egress.

The protein cargo of mosquito-derived EVs was instead investigated *ex vivo* in *A. aegypti*’s saliva and was shown to be altered following infection with DENV and to promote infection in human fibroblasts *in vitro*. Indeed, EVs released upon infection were loaded with pro-viral proteins including AAEL002675, proposed to act as an infection-enhancing protein (Gold et al., 2020). Mosquito saliva-derived EVs were also reported to contain subgenomic flaviviral RNA (sfRNA) (Yeh et al., 2023), likely enclosed within EVs, which enhanced infectivity in hepatoma cell line and primary fibroblasts *in vitro*. Importantly, this sfRNA exerted anti-immune effects through the inhibition of type I and III IFN responses to DENV2 infection (Yeh et al., 2023), potentially as part of an escape mechanism.

#### 2.1.2 Zika virus

Zika virus is a neurotropic flavivirus mainly transmitted via mosquito bite (*Aedes* spp.) (**Table 1**), although alternative transmission routes include sexual and vertical transmission (Gregory et al., 2017). ZIKV infection is mostly asymptomatic and self-limiting, however it can be responsible of neuropathology (Metzler and Tang, 2024) (**Table 2**). Of particular concern is transmission during pregnancy, since ZIKV can cause miscarriage or microcephaly in newborns, due to the development of brain abnormalities in fetus. In 2015 and 2016, ZIKV caused a number of outbreaks, especially in Latin America (Hills et al., 2017; Song et al., 2017) (https://www.who.int/news-room/fact-sheets/detail/zika-virus, last visited October 22nd, 2024). At present, there is no specific therapeutic intervention nor vaccine available to treat or prevent Zika infection, making this virus an important threat particularly for pregnant women in endemic areas.

ZIKV can infect different cell types including immature progenitors, astrocytes and microglia (**Table 1**) (Fikatas et al., 2021; Metzler and Tang, 2024). Even though the neurotropism of this virus is well established, probably representing a mechanism of immune evasion, the processes of crossing of the blood brain barrier (BBB) and the placental barrier are yet to be fully elucidated. Among different routes of cell entrance, ZIKV can invade target cells through a clathrin-dependent mechanism (Esteves et al., 2017), similarly to EVs uptake pathways (Ginini et al., 2022). This observation can have multiple implications, suggesting that this virus could exploit the EV-pathway to spread the infection. However, such a similarity could also represent a significant challenge in EV studies, particularly *in vitro*, as EVs might be difficult to distinguish from virions (**Figure 1**).

##### 2.1.2.1 EVs and *in vitro* ZIKV propagation

CNS cells - Due to the important neurological damages associated with ZIKV infection, it is not surprising that a number of studies investigated the mechanisms of neuro-invasion and neuro-pathogenesis. Different cell types of the CNS have been reported to release increased amount of EVs upon infection with ZIKV (**Supplementary Table S1**), including human primary astrocytes (Huang et al., 2018) and murine primary cortical neurons (Zhou et al., 2019), suggesting that they might be involved in viral replication and transmission. Indeed, reduced EVs release from ZIKV-infected human astrocytes via inhibition of sphingomyelinase-2 using a specific inhibitor (GW4869), resulted in decreased viral propagation and virion release (Huang et al., 2018). EVs from ZIKV-infected murine neurons were reported to contain viral RNA and viral proteins able to transmit the infection to naïve cells (Zhou et al., 2019), thus contributing to viral replication and transmission. Similar to the observations in human astrocytes (Huang et al., 2018), the release of EVs loaded with viral RNA and protein was reduced by sphingomyelinase-2 inhibition also in murine neurons (Zhou et al., 2019). The sphingomyelinase-2 pathway, involved in the production of ceramide and in the ESCRT-independent release of EVs (Shamseddine et al., 2015), could thus represent a novel potential therapeutic strategy to be further explored.

Other investigated brain-derived cell types include glioblastoma cells and human endothelial cells. The former were reported to release EVs with altered density and cargo upon ZIKV infection, and to carry viral genome, which induced a cytopathic effect when transferred to naïve cells (York et al., 2021). Likewise, hcMEC/D3 cells infected with ZIKV secreted EVs containing viral RNA, NS1 and E protein and were able to transfer this viral material to naïve cell types (Fikatas et al., 2021). EVs, similarly to the effect mediated by the whole virus, also induced early and transient perturbations in the monolayer integrity, indicating that they might contribute to the alterations of BBB permeability leading to brain invasion by ZIKV (Fikatas et al., 2021) (**Figure 2**). ZIKV-hcMEC-EVs also displayed an altered lipid cargo, compared to EVs from uninfected cells, suggesting a potential utility as biomarkers upon appropriate validation.

Embryonic and other cell types – Because of the important clinical consequences on fetuses, it is not surprising that EVs from embryonic cells are also raising attention (**Supplementary Table S1**), although due to ethical issues only a couple of studies have been published so far. Nonetheless, it has been shown that ZIKV infection altered the cargo of EVs released from embryonic cells. Infected macaque trophoblast stem cells (TSC) released EVs with altered mRNA, miRNA and proteins (Block et al., 2022); while ZIKV-infected human trophoblasts released EVs containing NS1 protein and an overall altered miRNA and protein cargo compared to mock-EVs, particularly enriched in mitochondrial proteins (Lee and Shin, 2023).

Lastly, human monocytes infected with ZIKV released EVs containing viral material, the transfer of which promoted viral transmission and infectivity in naïve cells, as well as monocyte differentiation towards an intermediate phenotype (Martinez-Rojas et al., 2024).

In agreement with observations for DENV, EVs derived from C6/36 cells infected with ZIKV were suggested to be involved in disease pathogenesis by modifying the host response (Martinez-Rojas et al., 2020). ZIKV-C6/36-EVs were in fact shown to be loaded with viral material (namely RNA and E protein) which could be transmitted and infect different types of naïve cells (including Vero E6, human monocytes and vascular endothelial cells). After internalization by target cells, they altered both immune and endothelial cells, by inducing monocyte differentiation and activation as well as alteration in endothelial permeability and inflammation, leading to a pro-inflammatory state (Martinez-Rojas et al., 2020) (**Figure 2**).

##### 2.1.2.2 Protective effects of EVs during ZIKV infection

In the context of ZIKV infection, host-derived EVs have been explored for their ability to mediate protective effects, thus preventing infection (**Figure 2**). Different body fluids have been shown to inhibit ZIKV infection in permissive cells *in vitro* and, interestingly, this ability was suggested to be mediated by the vesicular component (**Table 3**). Semen-EVs from healthy donors were reported to prevent ZIKV infection in epithelial cells of the genital tract (both cell lines and primary cells) (Muller et al., 2018; Wang et al., 2020). This inhibition was reported either to prevent the attachment to target cells, as shown by confocal fluorescence microscopy (Conzelmann et al., 2020), or to reduce the infection rate, as shown by the reduced viral load in cells simultaneously exposed to ZIKV and semen-EVs (Wang et al., 2020). EVs might thus be part of a defense mechanism that could contribute to the relatively limited rate of ZIKV sexual transmission despite the very high viral load found in semen in infected subjects (Muller et al., 2018).

Saliva-EVs were also proposed to be involved in a novel oral immune defense against ZIKV, as they inhibited infection in different types of target cells including simian Vero-E6, human cell lines (A549, Caco2, HeLa, HFF) and human primary cells (gingival fibroblasts) (Conzelmann et al., 2020). Saliva EVs blocked viral entry in host cells in a dose dependent fashion by inhibiting viral attachment, likely by occupying important sites for viral attachment on target cells. Interestingly, this anti-viral effect mediated by saliva-EVs was observed in different donors, even though the extent of the antiviral activity was donor-dependent (Conzelmann et al., 2020). The exact biological components responsible for this inhibition are yet to be determined and deserve further investigations. For instance, using artificial liposomes it has been proposed that lipids might mediate EV-membrane fusion with virions leading to loss of viral integrity (Wang et al., 2020), even though opposite results have also been reported (Conzelmann et al., 2020). Similarly to semen- (Muller et al., 2018) and saliva-EVs (Conzelmann et al., 2020), also EVs from urine and vaginal lavage have been described as able to inhibit ZIKV infection *in vitro*, yet to a lesser extent (Conzelmann et al., 2020).

Overall, these observations indicate that, in ZIKV, EVs from different body fluids might be important players of an innate defense mechanism as they can inhibit viral entry by avoiding or preventing attachment to target cells. EV bio-physical properties should now be investigated more comprehensively to identify the specific components responsible for this inhibitory effect and explore novel innovative therapeutic avenues.

##### 2.1.2.3 Therapeutic potential of EVs

EVs from different DENV- or ZIKV-infected cell types were reported to be loaded with viral material and to be able to transmit the infection to naïve cells (**Supplementary Table S1**). In contrast with this, CD9^+^-EVs released by ZIKV-infected HUVECs and enriched by immuno-capture, were reported to be unable to infect naïve cells, despite their cargo of viral RNA and proteins (Zhao et al., 2023). Interestingly, those EVs were particularly enriched with viral E protein on their surface and interacted with ZIKV-neutralizing antibodies, reducing the ADE effect due to competition for binding. ADE attenuation was observed both *in vitro* and *in vivo* - indicating a potential therapeutic or prophylaxis utility of E-coated flavivirus EVs. ZIKV E-coated-EVs also attenuated the ADE effect mediated by cross-reactive antibodies present in DENV patients’ serum (Zhao et al., 2023). As already mentioned, these observations contrast with the previously reported infectivity of flavivirus-EVs. At this stage it is difficult to establish whether these discrepancies are due to the very specific selection of CD9^+^ EVs by Zhao and colleagues (Zhao et al., 2023), or to the lack of purity of EVs preparations in the studies reporting EVs infective capacity, which could be contaminated with virions. It should however be mentioned that most of the reported studies included a number of controls in the EV preparation process, such as treatment with RNAse A or UV irradiation to reduce the possibility of contamination (**Supplementary Table S1**).

The therapeutic potential of EVs as highlighted by Zhao and colleagues should however be explored further, especially for diseases lacking of specific therapeutic intervention like arboviral infections. Among these, ZIKV presents the additional obstacle of treatment during pregnancy, which foresees the use of drugs able to cross the placental barrier, an immunologically privileged site. Previously, it was shown that IFITMs proteins inhibited ZIKV infection and ZIKV-induced cell death in different cell lines (Savidis et al., 2016), while the reduction in IFITM3 is associated with exacerbation of cell death in different cell types (Savidis et al., 2016; Monel et al., 2017). In DENV, EVs were proposed as a putative vehicle for delivering this antiviral factor to target cells (Zhu et al., 2015). It has thus been explored whether engineered EVs could be found useful in delivering IFITM3 to ZIKV infected fetus in mice (Zou et al., 2021). Remarkably, IFITM3-loaded EVs could cross the placental barrier effectively reducing viremia in different fetal organs, opening new avenues for anti-ZIKV treatment.

#### 2.1.3 West Nile virus

WNV is a flavivirus transmitted by *Culex* spp. mosquitos (**Table 1**). Humans and horses are considered dead-end hosts since they cannot transmit the infection due to the low circulating viral load, while birds represent the main reservoir (Campbell et al., 2002). In the majority of the cases, WNV gives rise to asymptomatic self-limiting infections, however in elderly or immunocompromised subjects severe forms can develop associated with neurological involvement (**Table 2**), as this virus can cross the BBB and infect neurons and glial cells (Winkelmann et al., 2016) (**Table 1**).

To the best of our knowledge, only a limited number of investigations focused on EVs in the context of WNV infection. WNV RNA was detected in EVs from human lung epithelial (A549) (Slonchak et al., 2019) and mouse neuroblastoma (N2a) (Zhou et al., 2018) infected cells, with the latter being able to transmit the infection to naïve cells (**Supplementary Table S1**). WNV altered host-RNA cargo (miRNAs, small-non coding RNAs and coding RNAs) of EVs released by A549 infected cells, particularly affecting RNA species associated with pro-inflammatory and anti-viral responses. Host-derived small RNAs released within EVs from WNV-infected cells, displayed immuno-stimulatory properties since they induced the expression of genes associated with the innate immune response in naïve host cells upon transfection (Slonchak et al., 2019).

#### 2.1.4 Japanese encephalitis virus

JEV is a flavivirus transmitted by *Culex* spp. mosquitoes, responsible for particularly severe neurological manifestations, including severe encephalitis in the human host (Turtle and Solomon, 2018; Banerjee and Tripathi, 2019) (**Tables 1**, **2**). Japanese encephalitis is associated with a high case fatality rate (up to 30%) and neurological sequelae in survivors (up to 50%) (Banerjee and Tripathi, 2019). Different vaccines have been developed and are currently employed in endemic countries to prevent the infection. Vaccination has mostly shown good efficacy, even though lower protection was reported in some areas (Turtle and Solomon, 2018; Banerjee and Tripathi, 2019). JEV pathology is characterized by neuro-inflammation associated with continued cytokine release following microglia activation, which also supports JEV replication (Banerjee and Tripathi, 2019).

Microglia cells are a well-known source of EVs within the CNS (Paolicelli et al., 2019), consequently they have also been investigated in the context of JEV. JEV-infected microglial cells were reported to release EVs containing increased amount of let-7a and let-7b miRNAs, the functional evaluation of which suggested their putative role in the neuro-inflammation associated with JEV infection (**Supplementary Table S1**). Indeed, their down-regulation led to attenuated pathology *in vitro*, while their over-expression was associated with an exacerbation of the neuronal damage (Mukherjee et al., 2019), supporting the role of microglia and their derived EVs in JEV neuro-pathogenesis.

### 2.2 Alphaviruses

#### 2.2.1 Chikungunya virus

Chikungunya virus is an alphavirus transmitted by *A. aegypti* and *A. albopictus* mosquitos (Bartholomeeusen et al., 2023) (**Tables 1**, **2**). Over the past years, a number of CHIKV outbreaks have been reported in endemic regions (i.e., Africa, South-East Asia, Latin America), but also in non-endemic countries (i.e., Italy and France) due to the presence of competent vectors (Angelini et al., 2008; Delisle et al., 2015). Recently, the first CHIKV vaccine (Ixchiq) has been approved by both FDA (November 2023) and EMA (May 2024) and is currently recommended for specific categories of subjects (Buerger et al., 2024; Freedman et al., 2024).

Very few direct observations have been collected regarding EVs and CHIKV. However, it has been shown that, similarly to other arboviruses, CHIKV-infected Vero-E6 cells released CD63^+^-EVs containing the entire viral genome as well as viral mRNA sequences. Importantly, these EVs were proposed to mediate viral transmission since they induced cytopathic effects in naïve Vero-E6 cells (**Supplementary Table S1**), even though a contamination of the EVs preparation with viral particles could not be excluded. As already shown for ZIKV and DENV, the inhibition of EV release by GW4869 was associated with reduced viral propagation *in vitro* (Le et al., 2022).

## 3 Tick-borne viruses

Compared to mosquito-borne flaviviruses, EVs are largely under-investigated in the context of tick-borne flaviviruses. A few reports are however available in the literature, proving that also these viruses might exploit EVs to transfer viral material from vector to host cells, as well as for their dissemination within the host.

### 3.1 Tick-borne encephalitis virus and Langat virus

Langat virus (LGTV) is a low pathogenic virus of the tick-borne encephalitis virus (TBEV) complex (Dobler, 2010) commonly used as a model to study TBEV. Vector (*Ixodes scapularis* ISE6) and human cells infected with LGTV secreted EVs containing viral RNA and proteins (Zhou et al., 2018). Using multiple *in vitro* models, those EVs were shown to be involved in the transmission of infective viral RNA at different levels, i.e., from tick cells to host keratinocytes and vascular endothelial cells; from brain endothelial cells to neurons; between neurons within the brain (**Supplementary Table S1**). This latter observation, led to the hypothesis that EVs might play a functional role in the neuro-pathogenesis associated with TBEV, as corroborated by the reduction in EVs viral load and infection capacity following GW4869 treatment (Zhou et al., 2018).

As reviewed by Butler et al., tick saliva was shown to contain EVs, the composition of which can influence pathogen (viruses and bacteria) transmission to the vertebrate host and modulate the host immune response (Butler et al., 2023). Briefly, EVs isolated from saliva and salivary glands from ixodid were reported to inhibit would healing, thus reducing the skin barrier protection and facilitating blood feeding (Zhou et al., 2020). Inhibiting vesiculation in the vector might thus represent a novel transmission blocking strategy (Sultana et al., 2024) and GW4869 was proposed as a novel candidate to block LGVT transmission and dissemination (Sultana et al., 2024).

### 3.2 Dabie bandavirus

Severe fever with thrombocytopenia syndrome (SFTS) virus, re-named Dabie bandavirus, is an emerging tick-borne virus, which is drawing attention in light of the recent geographic expansion of ticks responsible for its transmission (Kim and Park, 2023). Firstly identified in China in 2009, the virus has then spread within China and to East Asian countries (Yu et al., 2011; Kim and Park, 2023). Due to its relatively recent emergence, many aspects of the biology of this virus are yet to be fully described, making our knowledge still limited. However, efforts have already been made to characterize its EVs (Silvas et al., 2016) showing that SFTS-infected HeLa cells released CD63^+^-EVs containing the viral non-structural protein (NSs). In agreement with numerous observations reported for other arboviruses, STSF virus-EVs were shown to be loaded with SFTS infectious virions and to be able to sustain viral replication upon delivery to target naïve cells (Silvas et al., 2016) (**Supplementary Table S1**), supporting once more the hypothesis that arboviruses hijack the secretory endosomal pathway to sustain their transmission.

## 4 Current challenges and future perspectives

Different factors, including climate change and globalization, are re-shaping the geographical distribution of vector-borne viruses, making these infections important emerging or re-emerging health threats. It is thus not surprising that the scientific community has engaged in fighting these diseases from different angles, increasing our knowledge of these viruses and of the pathology they cause. In this context, it has become clear that EVs play a dual role during arboviral infections, by either promoting or preventing infection. Viruses are in fact able to manipulate EV release from host cells to their own advantage as a mechanism to sustain viral transmission while escaping the host immune response. Nonetheless, EVs shed by immune cells responding to these invading pathogens are essential in supporting antiviral responses (Martin et al., 2023).

This dual role amplifies the potential clinical applications of these EVs for arboviral infections, spanning from disease biomarkers, control strategies and therapeutic intervention, without forgetting their utility in better understanding the disease pathobiology. Nevertheless, as also highlighted in the present manuscript, the potential role of EVs as infection facilitators is probably receiving more attention compared to EVs as mediators of antiviral response. A more comprehensive investigation of the latter should thus be envisaged.

EVs have a strong potential to be used as liquid biopsies. They are in fact generally present at high concentration in numerous body fluids and their concentration can be strongly altered during pathological processes. These properties make EVs very promising candidate biomarkers. In addition to alterations in EVs quantities, modulations in their cargo of proteins, nucleic acids, metabolites or lipids might serve as diagnostic or prognostic biomarkers, especially since biomolecules enclosed within EVs are considered to be more stable compared to freely circulating ones.

As described in this review manuscript, alterations in EVs cargo, particularly affecting miRNA, have been investigated in association with arboviral infections. Notably, they have been suggested as predictive markers of disease progression towards severe forms for DENV and WNV (Punyadee et al., 2015; Martins et al., 2018; Slonchak et al., 2019; Pradhan et al., 2023), or as markers of placental involvement in ZIKV infection (Block et al., 2022).

Most vector-borne viruses have the ability to invade and infect the CNS, giving rise to neuro-inflammation accompanied by clinical manifestations of different entities. These can span from mild self-limiting manifestations to severe, and potentially fatal, manifestations like viral encephalitis, meningitis and acute flaccid paralysis (Alissa et al., 2024) (**Table 2**). EVs might have an interesting potential as early prognostic markers of neurological involvement, since they were suggested to mediate the neuro-inflammation associated with neurotropic arboviral infections (Huang et al., 2018; Zhou et al., 2018; Mukherjee et al., 2019; Zhou et al., 2019; Mishra et al., 2020; Fikatas et al., 2021) and had already been associated with other CNS infections, like human African trypanosomiasis (Dozio et al., 2019) and cerebral malaria (Debs et al., 2019).

During arboviral infections, EVs can be released by infected and un-infected cells from both the human host and the vector. EVs released by vector- or host-infected cells have been reported to enhance viral transmission basically in all the diseases here reported. This property could be particularly interesting in the context of a transmission blocking strategy, since it has been shown that blocking tetraspanins to inhibit vesicle biogenesis in infected cells reduces viral transmission (Huang et al., 2018; Zhou et al., 2019; Sultana et al., 2024). A more in-depth comprehension of the mechanisms of interaction between the virus and the host might thus reveal targets for the development of novel therapeutic or vaccine strategies. Intrinsic EVs properties make them very promising therapeutic tools. Indeed, EVs are naturally able to cross physiological barriers gaining access to immune privileged sites. Moreover, due to their role in intercellular communication and they lower immunogenicity compared to cell-based therapies, EVs can be engineered and exploited to deliver biological active compounds to target sites (Zhao et al., 2024). Although EV-therapeutic potential was investigated more in depth in other pathological conditions, a couple of studies focusing on arboviral infections were also published. In particular, it was reported that IFITM3-containing EVs display anti-viral properties against DENV and ZIKV (Zhu et al., 2015; Zou et al., 2021). Since EVs are being revealed as promising vaccination and therapeutic delivery platforms, their more extensive investigation might contribute in revealing novel control strategies for arboviral infections.

Although it is now clear that EVs might have several implications and future applications in the context of arboviral infections, a number of important aspects still need to be further explored before they can find a clinical utility.

The enumeration and characterization of EVs in patients’ body fluid still present important technical and biological variabilities. Pre-analytical sample management (including sample collection, storage and transportation) and the methods selected for their enrichment can profoundly affect the results of their downstream analysis and characterization. To overcome these limitations, enrichment-free approaches based on the direct quantification of EVs in the biological fluid of interest should be envisaged. Researchers should also comply with the guidelines provided by the ISEV (Lotvall et al., 2014; Thery et al., 2018; Welsh et al., 2024) in order to standardize the analytical procedures. These factors might partly explain the disagreement between some of the results reported by different studies investigating EVs as DENV prognostic markers. Actually, the lack of reporting accuracy of scientific studies is an important drawback in EVs translation into clinical practice. EVs are highly heterogeneous entities, which might be involved in different biological processes depending on their surface composition, cargo of biomolecules, size and biogenesis. The compliance with good reporting practice and ISEV guidelines is thus paramount to ensure a proper characterization of the studied EV population, reproducibility of the results and their accurate evaluation in the clinical context.

One additional challenge when working with EVs in the field of virology is the difficulty in distinguishing between vesicles and viral particles (**Figure 1**), since they share many bio-physical properties and, in some instances, the mechanisms of biogenesis (Nolte-’t-Hoen et al., 2016; Kutchy et al., 2020; Rey-Cadilhac et al., 2023). This is particularly relevant in those studies evaluating the ability of EVs to transfer viral genomes and to contribute to viral propagation to uninfected cells. Indeed, it could be hypothesized that some of the functional properties conferred to EVs derived from infected cells, are actually mediated by viral particles that “contaminate” the EVs preparation, rather than by the EVs themselves. In order for these observations to be reliable, it is essential to demonstrate the actual association of this genetic infective material with EVs rather that the presence of co-enriched virion particles. In the attempt to try to discern this intricate problem, Ishikawa and colleagues employed sub-genomic replicons, which are able to replicate within the host cell but unable to generate replication-competent viruses (Ishikawa et al., 2024). EVs released by cells harboring sub-genomic replicons were shown to contain viral RNA and proteins, and to be able to transfer this RNA to recipient cells, in which it was then replicated (Ishikawa et al., 2024). Importantly, this property was demonstrated with two different flaviviruses, i.e., DENV and JEV, and with a number of distinct cell types from different host species including human (HeLa – cervix adenocarcinoma, SK-N-SH – neuroblastoma, HepG2 – hepatoma; K562 – leukaemia), monkey (Vero – kidney), hamster (BHK – kidney), pig (PK15 – kidney) and *A. albopictus* (C6/36). Most of the studies dealing with EVs ability to transfer infective viral material employed some “control strategies” such as treatment with RNase A or TritonX-100 to prove that the infective material is actually the EV inner cargo and not an extra-vesicular contamination. In addition to this, the observations reported by Ishikawa and colleagues further support the importance of EVs as an alternative mechanism for inter-cellular dissemination of viral genome of arthropod-borne viruses while escaping neutralizing antibodies.

The apparent discrepancies regarding the potential functional role of EVs during arboviral infections reported in different studies are probably revealing the multifaceted role of these elements. Indeed, very likely there is not a univocal cause-effect relationship between arboviral infections and EVs but rather a dynamic association. As a consequence, the different properties revealed by different studies should be further investigated exploiting the ever-growing field of nanotechnologies. Similarly to what was done for ZIKV (Wang et al., 2020), the production of artificial nanoparticles replicating one or more of the bio-physical properties observed in the biological context might represent a powerful validation strategy. Despite a number of obstacles still need to be overcome to get EVs research closer to clinical applications, their potential as novel tools to control the spread of arboviral infections is now evident. Translational research should now focus on the comprehensive evaluation and validation of this sub-micron elements as targeted delivery systems of antiviral factors (e.g., IFITM3), as prognostic or severity markers or as targets of novel therapeutic options.

## Glossary

DENV: Dengue virus
ZIKV: Zika virus
CHIKV: Chikungunya virus
YFV: Yellow fever virus
OROV: Oropouche virus
RVFV: Rift Valley fever virus
WNV: West Nile virus
TOSV: Toscana virus
USUV: Usutu virus
TBEV: Tick borne encephalitis virus
JEV: Japanese encephalitis virus
CCHFV: Crimean-Congo hemorrhagic fever
EVs: extracellular vesicles
MVB: multivesicular body
ISEV: International Society for Extracellular Vesicles
ESCRT: endosomal sorting complexes required for transport
BBB: blood brain barrier
HIV: human immunodeficiency virus
HTLV-1: human T-lymphotropic virus
HCV: hepatitis C virus
WHO: World Health Organization
ADE: antibody dependent enhancement
IL-1β: interleukin 1 beta
CRP: C-reactive protein
SAA: serum amyloid A
sVCAM-1: soluble vascular cell adhesion molecule 1
sICAM-1: soluble intercellular adhesion molecule 1
CLEC2: C-type lectin receptor 2
NET: neutrophil extracellular trap
AnV: annexin V
NS1: nonstructural protein 1
PD-L1: programmed death-ligand 1
PD-1: programmed cell death-1
USP33: ubiquitin-specific peptidase 33
IFN: interferon
HBV: hepatitis B virus
IFITM3: interferon-inducible transmembrane protein 3
DHF: dengue hemorrhagic fever or severe dengue
DF: dengue fever or mild-dengue
TNFα: tumor necrosis factor alpha
sfRNA: subgenomic flaviviral RNA
CNS: central nervous system
FDA: food and drug administration
EMA: European medicines agency
LGTV: Langat virus
SFTS: severe fever with thrombocytopenia
HepG2: human liver epithelial cells
HUVEC: human primary umbilical vein endothelial cells
PBMCs: peripheral blood mononuclear cells
THP-1: human monocytic cell line
HEK2937: human embryonic kidney epithelial cell line
RBC: red blood cells
DCs: dendritic cells
hcMEC/D3: human brain microvascular endothelial cells
A549: human lung epithelial cells
Caco2: human colorectal epithelial cell line
HeLa: human cervical epithelial cells
HFF: human fibroblasts
Vero-E6: green monkeykidney epithelial cells
