# Supplementary material for: Extracellular vesicles in arbovirus infections: from basic biology to potential clinical applications
Source: Front Cell Infect Microbiol. 2025 Apr 28;15:1558520. doi: 10.3389/fcimb.2025.1558520 (PMC12066795; doi:10.3389/fcimb.2025.1558520)
Supplement: Supplementary file 1 [file DataSheet1.pdf]

Extracellular vesicles in arbovirus infections: from basic biology to potential clinical applications

Natalia Tiberti[1*], Concetta Castilletti[1], Federico Giovanni Gobbi[1,2]

Supplementary Material

Supplementary Table S1. Summary of the most relevant observations regarding EVs released by *in vitro* infected cells and their functional properties on target cells

Infected cell type	Pathogen strain	EV enrichment method	Properties of EVs from infected cells	Cargo of viral material	Effects on naive cells	Controls in EV preparation	Ref.
**DENV**							
Platelets	DENV-2 (16881)	Low speed centrifugation	Increased release of EVs and IL-1β$^+$EVs	nd	Increased endothelial (HMEC-1) permeability	nd	(1)
Platelets (DENV patients)	na	UC	Increased secretion of EVs	nd	Loss of adhesion molecules and increased vascular inflammation in HUVECs	nd	(2)
Platelets	DENV2 (PL046 and New Guinea C-N)	Low speed centrifugation + UC	Upregulated CD62p$^+$/CD41$^+$ and CD63$^+$/CD41$^+$ EVs	nd	Induction of NET formation in neutrophils; Increased vascular permeability in HMEC-1	nd	(3)
HepG2 HUVECs	DENV2 (16881)	Differential centrifugation	Increased release of AnV$^+$ EVs	E and NS1 on the surface	nd	nd	(4)
THP1 HEK293T (DENV-NS1-transfected)	DENV2 (New Guinea C)	Salting out	Increased release of EVs enriched with miR-148a	nd	Enhancement of neuro-inflammation in micgroglial cells (CHEM3)	EVs from HEK293T transfected with DENV-NS1 gene	(5)
Monocyte-derived DCs	DENV3 (5532 and 290)	UC	Altered miRNA and mRNA cargo	Viral RNA or viral particles	Infective in C6/36 cells	4G2-Anti-dengue E protein antibody	(6)
U937	DENV2 (Colombian isolated)	UC + sucrose gradient + CD36 IP	Heterogeneous EV population, protein cargo involved in	NS3 protein	Endothelial activation (EA.hy926)<br>Non-infective in epithelial cells (LLC-MK2, monkey)	UV irradiation	(7)

			the immune response				
C6/36 (*A. albopictus*) Aag-2 (*A. aegypti*)	DENV2 (TVP2176, New Guinea C) DENV3 (BID-V1619/2005)	OptiPrep DG or exosome isolation kit or UC	EVs size range of 30-250 nm; increased amount of small EVs	Viral RNA (full-length genome), E and NS1 proteins	Infective in C6/36 cells, Vero E6, mouse DCs, human-skin keratinocytes and HUVECs	RNase A and Triton X-100 4G2-antibody bead binding assay	(8)
C6/36 (*A. albopictus*)	DENV2 (New Guinea C)	UC + exosome isolation kit + $CD9^+$ selection	Larger size EVs	Virus-like particles	Infective in C6/36	Immuno-magnetic bead affinity purification (CD9)	(9)
ATC-10 (*A. aegypti)*	DENV2 (New Guinea C)	Exosome isolation kit	Altered protein cargo, loaded with AAEL00275 protein	nd	Infection enhancing capacity of AAEL00275 in human primary dermal fibroblasts	nd	(10)
A. albopictus, saliva	DENV2 (New Guinea C)	None	EV-like particles of 100-500 nm	3'UTR sequences, Subgenomic RNA	nd	Triton X-100, RNase A and Proteinase K	(11)
**ZIKV**							
Primary astrocytes	ZIKV MR766 and PRVABC59	UC	Increased release of EVs	nd	nd	nd	(12)
Primary cortical neurons (mouse)	ZIKV PRVABC59	UC or DG	Increased release of EVs	RNA, E protein	Infective in neuronal cells	RNase A, neutralizing antibodies	(13)
SNB-19	ZIKV PRVABC59	UC + gradient purification	Increased release of EV with wide size range and density	RNA, E protein	Infective in Vero E6	RNase A and Triton X-100 treatment	(14)
hcMEC/D3	ZIKV PRVABC59	Differential centrifugation + ultra-filtration + DG	Altered lipid cargo	RNA, E and NS1 proteins	Transfer of viral material to hcMEC/D3 and gliobastoma cells	RNase A treatment	(15)
Early gestation trophoblasts (TSCs, macaque)	ZIKV DAK AR 41524 NR-50338 ("DAKAR")	Size exclusion chromatography	Altered mRNA, miRNA and protein cargo	nd	nd	nd	(16)

JEG-3 cells	ZIKV MR766 and PRVABC59	Exo-Spin kit	Altered cargo of miRNA and proteins	NS1 protein	nd	nd	(17)
THP1	ZIKV MR766	UC	na	E and NS1 proteins, virus like particles	Infective in Vero E6 and monocytes. Monocyte activation and differentiation	UV irradiation, RNase A	(18)
C6/36 (*A. albopictus*)	ZIKV MR766	UC	Release of medium (AnV^+) and small ($CD63^+$) EVs	Viral RNA and E protein	Infective in Vero E6, THP-1, HMEC-1. Promotion of monocyte differentiation and activation; alteration of endothelial permeability and inflammation	UV irradiation, RNase A	(18)
HUVECs	Stabilized infectious cDNA clone pZL1	UC + CD9 immuno-capture	E protein on EVs surface and binding to neutralizing antibodies	Capsid, prM, E, NS1, and NS5 proteins, viral RNA	Non-infective in Vero-E6	EV affinity enrichment	(19)
**WNV**							
A549	WNV Kunjin	ExoQuick-TC	Altered miRNAs, RNAs, ncRNAs cargo	nd	nd	Treatment with heat (56°C) and RNase A	(20)
N2a (mouse)	WNV CT2741 wild-type	OptiPrep DG	na	RNA	Infective in N2a	nd	(21)
**JEV**							
N9 Microglial cells (mouse)	JEV GP78	Exosome isolation reagent	Increased let-7a and let-7b miRNAs	nd	Accelerate neuronal damage in N2a cells	nd	(22)
**CHIKV**							
Vero E6 (green monkey)	CHIKV ROSS C6D4	Total exosome isolation kit + CD63 isolation kit	Release of small EVs	E1 mRNA, Viral genome	Infective in Vero E6 cells	RNase A and Triton X-100	(23)
**LGTV (as model for TBEV)**							
ISE6 (*I. scapularis*)	LGTV LGT-TP21	OptiPrep DG	Size range: 30-200nm	E and NS1 proteins, viral RNA	Infective in human keratinocytes and HUVEC	Triton X-100, 4G2 antibody	(21)
bEnd.3 (mouse)	LGTV LGT-TP21	OptiPrep DG	Size range: 30-200nm	Viral RNA	Infective in N2a cells	nd	(21)

N2a (mouse)	LGTV LGT-TP21	OptiPrep DG	Size range: 30-200nm	E protein, viral RNA	Infective in N2a cells	Proteinase K and RNase A	(21)
**SFTS virus**							
HeLa	SFTS virus	UC + CD63 IP	na	NSs protein, virions	Infective in HeLa cells	IP, negative selection	(24)

HepG2: liver epithelial cells; HUVECs: primary umbilical vein endothelial cells; THP1: monocyte cell line; HEK293T: embryonic kidney epithelial cell line; DCs: dendritic cells; hcMEC/D3: human brain microvascular endothelial cells; U937: macrophages; SNB-19: gliobastoma cell line; JEG-3: trophoblasts; A549: lung epithelial cells; N2a: murine neuroblastoma cell line; Vero E6: green monkey kidney epithelial cells; HeLa: cervical epithelial cells; bEnd.3: murine brain-microvascular endothelial cells. All cells are of human origin, unless otherwise specified.
UC: ultracentrifugation; DG: density gradient; IP: immunoprecipitation; na: not available; nd: not determined.

References

1. Hottz ED, Lopes JF, Freitas C, Valls-de-Souza R, Oliveira MF, Bozza MT, et al. Platelets mediate increased endothelium permeability in dengue through NLRP3-inflammasome activation. Blood. 2013 Nov 14;122(20):3405-14. PubMed PMID: 24009231. Pubmed Central PMCID: 3829114.

2. Vedpathak S, Sharma A, Palkar S, Bhatt VR, Patil VC, Kakrani AL, et al. Platelet derived exosomes disrupt endothelial cell monolayer integrity and enhance vascular inflammation in dengue patients. Frontiers in immunology. 2023;14:1285162. PubMed PMID: 38235130. Pubmed Central PMCID: 10791899.

3. Sung PS, Huang TF, Hsieh SL. Extracellular vesicles from CLEC2-activated platelets enhance dengue virus-induced lethality via CLEC5A/TLR2. Nature communications. 2019 Jun 3;10(1):2402. PubMed PMID: 31160588. Pubmed Central PMCID: 6546763.

4. Punyadee N, Mairiang D, Thiemmeca S, Komoltri C, Pan-Ngum W, Chomanee N, et al. Microparticles provide a novel biomarker to predict severe clinical outcomes of dengue virus infection. Journal of virology. 2015 Feb;89(3):1587-607. PubMed PMID: 25410854. Pubmed Central PMCID: 4300736.

5. Mishra R, Lahon A, Banerjea AC. Dengue Virus Degrades USP33-ATF3 Axis via Extracellular Vesicles to Activate Human Microglial Cells. Journal of immunology. 2020 Oct 1;205(7):1787-98. PubMed PMID: 32848034.

6. Martins ST, Kuczera D, Lotvall J, Bordignon J, Alves LR. Characterization of Dendritic Cell-Derived Extracellular Vesicles During Dengue Virus Infection. Frontiers in microbiology. 2018;9:1792. PubMed PMID: 30131785. Pubmed Central PMCID: 6090163.

7. Velandia-Romero ML, Calderon-Pelaez MA, Balbas-Tepedino A, Marquez-Ortiz RA, Madronero LJ, Barreto Prieto A, et al. Extracellular vesicles of U937 macrophage cell line infected with DENV-2 induce activation in endothelial cells EA.hy926. PloS one. 2020;15(1):e0227030. PubMed PMID: 31910224. Pubmed Central PMCID: 6946137.

8. Vora A, Zhou W, Londono-Renteria B, Woodson M, Sherman MB, Colpitts TM, et al. Arthropod EVs mediate dengue virus transmission through interaction with a tetraspanin domain containing glycoprotein Tsp29Fb. Proceedings of the National Academy of Sciences of the United States of America. 2018 Jul 10;115(28):E6604-E13. PubMed PMID: 29946031. Pubmed Central PMCID: 6048473.

9. Reyes-Ruiz JM, Osuna-Ramos JF, De Jesus-Gonzalez LA, Hurtado-Monzon AM, Farfan-Morales CN, Cervantes-Salazar M, et al. Isolation and characterization of exosomes released from mosquito cells infected with dengue virus. Virus research. 2019 Jun;266:1-14. PubMed PMID: 30930201.

10. Gold AS, Feitosa-Suntheimer F, Araujo RV, Hekman RM, Asad S, Londono-Renteria B, et al. Dengue Virus Infection of Aedes aegypti Alters Extracellular Vesicle Protein Cargo to Enhance Virus Transmission. International journal of molecular sciences. 2020 Sep 10;21(18). PubMed PMID: 32927629. Pubmed Central PMCID: 7555558.

11. Yeh SC, Strilets T, Tan WL, Castillo D, Medkour H, Rey-Cadilhac F, et al. The anti-immune dengue subgenomic flaviviral RNA is present in vesicles in mosquito saliva and is associated with increased infectivity. PLoS pathogens. 2023 Mar;19(3):e1011224. PubMed PMID: 36996041. Pubmed Central PMCID: 10062553.

12. Huang Y, Li Y, Zhang H, Zhao R, Jing R, Xu Y, et al. Zika virus propagation and release in human fetal astrocytes can be suppressed by neutral sphingomyelinase-2 inhibitor GW4869. Cell discovery. 2018;4:19. PubMed PMID: 29707233. Pubmed Central PMCID: 5913238.

13. Zhou W, Woodson M, Sherman MB, Neelakanta G, Sultana H. Exosomes mediate Zika virus transmission through SMPD3 neutral Sphingomyelinase in cortical neurons. Emerging microbes & infections. 2019;8(1):307-26. PubMed PMID: 30866785. Pubmed Central PMCID: 6455149.

14. York SB, Sun L, Cone AS, Duke LC, Cheerathodi MR, Meckes DG, Jr. Zika Virus Hijacks Extracellular Vesicle Tetraspanin Pathways for Cell-to-Cell Transmission. mSphere. 2021 Jun 30;6(3):e0019221. PubMed PMID: 34190582. Pubmed Central PMCID: 8265634.

15. Fikatas A, Dehairs J, Noppen S, Doijen J, Vanderhoydonc F, Meyen E, et al. Deciphering the Role of Extracellular Vesicles Derived from ZIKV-Infected hcMEC/D3 Cells on the Blood-Brain Barrier System. Viruses. 2021 Nov 25;13(12). PubMed PMID: 34960632. Pubmed Central PMCID: 8708812.

16. Block LN, Schmidt JK, Keuler NS, McKeon MC, Bowman BD, Wiepz GJ, et al. Zika virus impacts extracellular vesicle composition and cellular gene expression in macaque early gestation trophoblasts. Scientific reports. 2022 May 5;12(1):7348. PubMed PMID: 35513694. Pubmed Central PMCID: 9072346.

17. Lee JK, Shin OS. Zika virus modulates mitochondrial dynamics, mitophagy, and mitochondria-derived vesicles to facilitate viral replication in trophoblast cells. Frontiers in immunology. 2023;14:1203645. PubMed PMID: 37781396. Pubmed Central PMCID: 10539660.

18. Martinez-Rojas PP, Monroy-Martinez V, Agredano-Moreno LT, Jimenez-Garcia LF, Ruiz-Ordaz BH. Zika Virus-Infected Monocyte Exosomes Mediate Cell-to-Cell Viral Transmission. Cells. 2024 Jan 12;13(2). PubMed PMID: 38247836. Pubmed Central PMCID: 10814160.

19. Zhao F, Xu Y, Liu N, Lv D, Chen Y, Liu Z, et al. Extracellular vesicles from Zika virus-infected cells display viral E protein that binds ZIKV-neutralizing antibodies to prevent infection enhancement. The EMBO journal. 2023 Mar 15;42(6):e112096. PubMed PMID: 36734074. Pubmed Central PMCID: 10015360.

20. Slonchak A, Clarke B, Mackenzie J, Amarilla AA, Setoh YX, Khromykh AA. West Nile virus infection and interferon alpha treatment alter the spectrum and the levels of coding and noncoding host RNAs secreted in extracellular vesicles. BMC genomics. 2019 Jun 10;20(1):474. PubMed PMID: 31182021. Pubmed Central PMCID: 6558756.

21. Zhou W, Woodson M, Neupane B, Bai F, Sherman MB, Choi KH, et al. Exosomes serve as novel modes of tick-borne flavivirus transmission from arthropod to human cells and facilitates dissemination of viral RNA and proteins to the vertebrate neuronal cells. PLoS pathogens. 2018 Jan;14(1):e1006764. PubMed PMID: 29300779. Pubmed Central PMCID: 5754134.

22. Mukherjee S, Akbar I, Kumari B, Vrati S, Basu A, Banerjee A. Japanese Encephalitis Virus-induced let-7a/b interacted with the NOTCH-TLR7 pathway in microglia and facilitated neuronal death via caspase activation. Journal of neurochemistry. 2019 May;149(4):518-34. PubMed PMID: 30556910.

23. Le BCT, Burassakarn A, Tongchai P, Ekalaksananan T, Aromseree S, Phanthanawiboon S, et al. Characterization and Involvement of Exosomes Originating from Chikungunya Virus-Infected Epithelial Cells in the Transmission of Infectious Viral Elements. International journal of molecular sciences. 2022 Oct 11;23(20). PubMed PMID: 36292974. Pubmed Central PMCID: 9603488.

24. Silvas JA, Popov VL, Paulucci-Holthauzen A, Aguilar PV. Extracellular Vesicles Mediate Receptor-Independent Transmission of Novel Tick-Borne Bunyavirus. Journal of virology. 2016 Jan 15;90(2):873-86. PubMed PMID: 26512089. Pubmed Central PMCID: 4702678.
